# Retroperitoneal Hydropic Leiomyoma Mimicking an Ovarian Cyst

**DOI:** 10.1155/2022/2012376

**Published:** 2022-08-04

**Authors:** Pei-Hsuan Lai, Dah-Ching Ding

**Affiliations:** ^1^Department of Obstetrics and Gynecology, Hualien Tzu Chi Hospital, Buddhist Tzu Chi Foundation, Tzu Chi University, Hualien, Taiwan; ^2^Institute of Medical Sciences, College of Medicine, Tzu Chi University, Hualien, Taiwan

## Abstract

Leiomyoma is the most common benign neoplasm of the reproductive organs in women. Retroperitoneal hydropic leiomyoma is rare type of myoma. Herein, we present the case of a 46-year-old (gravida 0) woman with retroperitoneal hydropic leiomyoma that was preoperatively diagnosed as an ovarian cyst. Transvaginal sonography and abdominal computed tomography revealed a mass, measuring 8.1 × 3.8 cm, with solid and cystic components in the right pelvic cavity. The patient underwent laparoendoscopic single-site surgery for the tumor excision. During the surgery, a retroperitoneal cystic tumor was resected from the right retroperitoneal cavity. Histopathologic and microscopic examinations revealed a hydropic leiomyoma with infarction. This case is impressive because of the rare location and hydropic degeneration of the leiomyoma. Furthermore, it mimicked an ovarian cyst at its initial presentation making accurate diagnosis difficult. By warning of this case, surgeons can recognize the disease entities and provide the necessary treatment.

## 1. Introduction

Leiomyomas, also known as myomas or fibroids, are the most common benign neoplasms of the reproductive organs in women of reproductive age [1]. These tumors are of mesenchymal origin and predominantly composed of smooth muscle cells separated by variable amounts of fibrous connective tissue. They are mostly found within the uterine cavity (submucosal), myometrium (intramural), or under the uterine serosa (subserosal). Extrauterine retroperitoneal growth pattern in leiomyoma is rare, which makes its diagnosis challenging [2]. Various types of degenerations may occur as the leiomyoma enlarges and outgrows its blood supply, resulting in altered sonographic appearances [3]. These degenerations include hyaline or myxoid, cystic, red, and calcification [3].

Hydropic leiomyoma (HLM) is a rare variant of uterine leiomyoma with characteristic features such as zonal distribution of edema, increased vascularity, and arrangement of tumor cells in nodules or cords [4]. Detailed case studies on HLM are lacking. Here, we describe a case of retroperitoneal HLM that was preoperatively diagnosed as an ovarian cyst on the basis of clinical manifestations and ultrasound imaging.

## 2. Case Report

**Figure 1:**
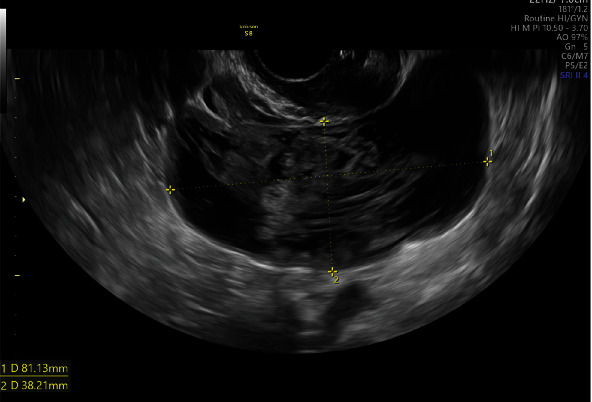
The transvaginal sonography revealed a right adnexal complex mass measuring 8.1 × 3.8 cm that did not resolve after three months of expectant management.

**Figure 2:**
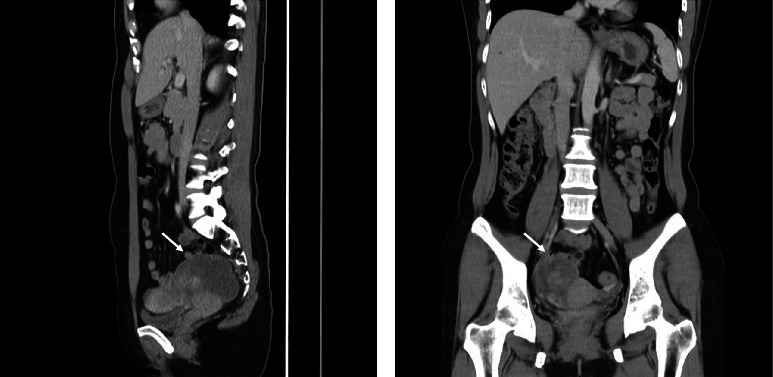
Abdominal computed tomography of the tumor. The tumor was with solid and cystic components in the right pelvic cavity. (a) Sagittal view (arrow), (b) coronal view (arrow).

A 46-year-old gravida 0 woman visited our outpatient clinic to consult for a hydrosalpinx that she incidentally found a month ago. She was asymptomatic, without any complaints or discomfort. There were neither significant comorbidities nor a family history of gynecological diseases. Transvaginal sonography revealed a right adnexal complex mass measuring 8.1 × 3.8 cm that did not resolve after three months of expectant management (Figure 1). Her cancer antigen 125 (CA 125) and 19-9 as well as carcinoembryonic antigen levels were within normal limits. Abdominal computed tomography (CT) revealed a mass with solid and cystic components in the right pelvic cavity (Figure 2). The anteverted uterine body was compressed and displaced to the left side. Enlarged lymph nodes were absent in the pelvic cavity and para-aortic regions.

**Figure 3:**
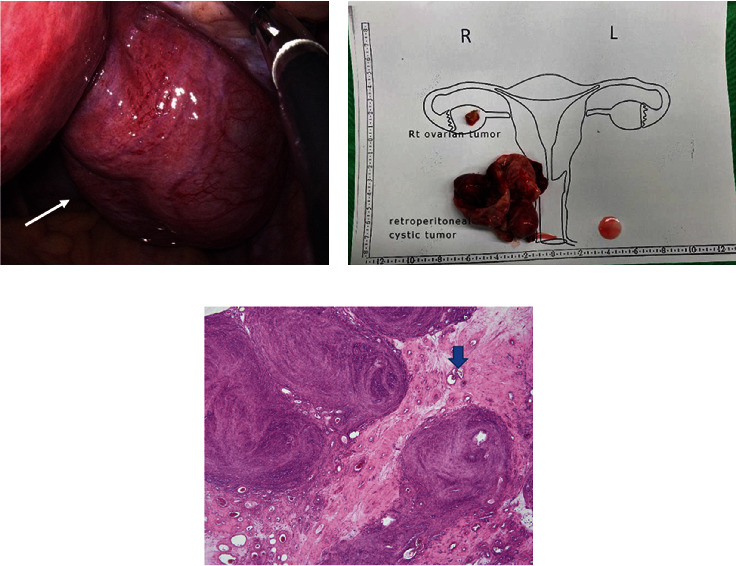
Laparoscopic view, gross, and microscopic picture of the tumor. (a) During the surgery, a retroperitoneal cystic tumor measuring 8 × 4 cm was found located in the right pelvic cavity (arrow). (b) The tumor was grayish-white and grossly elastic. (c) Microscopically, the tumor revealed leiomyoma with infarction and cystic degeneration (arrow).

The patient underwent a laparoendoscopic single-site surgery for exploration and excision of the tumor. A possible staging surgery was planned in case of malignancy. During the surgery, a retroperitoneal cystic tumor located in the right retroperitoneal cavity was found (Figure 3(a)). After resecting the tumor, the frozen sections revealed a leiomyoma. The tumor was grayish-white and grossly elastic (Figure 3(b)). Microscopic examinations revealed infarction and hydropic degeneration of the leiomyoma (Figure 3(c)). The tumor was moderately cellular without nuclear atypia or coagulative necrosis, and mitotic figures were rare. The definitive diagnosis was HLM. The patient recovered from the surgery uneventfully. A follow-up transvaginal sonography revealed no remarkable lesions. Further recurrence has not been reported so far.

## 3. Discussion

Retroperitoneal tumors can be primary or metastatic. Primary retroperitoneal tumors are extremely rare and usually arise from the tissues that form the retroperitoneal spaces rather than the retroperitoneal organs [5]. Malignant tumors of the retroperitoneum are approximately fourfold more frequent than the benign lesions. In a multi-institutional cohort study of 167 Japanese patients from 2000 to 2012, Fujimoto et al. concluded that liposarcomas were the most common type, followed by schwannomas, paragangliomas, and leiomyosarcomas [6]. To evaluate the possibility of malignancies, laboratory and radiologic investigations followed by an accurate histopathological examination are necessary.

Retroperitoneal leiomyoma is an even rarer condition with an undetermined incidence whose pathogenesis and biology remain controversial. The presenting symptoms usually arise unspecifically from the compression of adjacent structures and may be dependent on the size and location of the tumor and its relation with the surroundings [7]. A literature review identified 105 reported cases of retroperitoneal leiomyoma from 1941 to 2007, wherein 25% of the patients were asymptomatic, 31.3% experienced abdominal fullness, 18.8% had urinary symptoms, 18.8% had weight loss, and 18.8% had pelvic pain [2]. Pelvic mass was noted on palpation for almost 90% of the patients [2]. However, the patient in the current study had no tumor-related symptoms whatsoever.

Typically, ultrasonography of uterine leiomyoma reveals a well-defined, solid, isoechoic, or hypoechoic mass compared to the normal myometrium. Leiomyomas with cystic degeneration may have a complex appearance, and color Doppler ultrasound typically shows circumferential vascularity [8]. However, when it comes to masses located at the retroperitoneum, the preoperative diagnosis is challenging due to the wide variety of imaging characteristics of retroperitoneal tumors along with the difficulty in distinguishing them from the ovaries [2]. The anatomic origin of the mass is hard to discern even with CT, magnetic resonance image (MRI), or both [2]. Although MRI may currently be the most useful imaging modality, it requires further advancements [9]. Both pelvic ultrasound and CT scan revealed ovarian cyst-like lesions in the patient of this study.

Tumor markers, especially the CA-125, are usually elevated in patients with uterine leiomyomas. Raised CA-125 levels are considered to be caused by peritoneal irritation, secondary to the larger myoma size (>5 cm) [10]. For preoperative evaluation, we checked for tumor markers that were not significantly elevated.

On gross examination, retroperitoneal leiomyomas typically present as circumscribed, oval to spherical, white to gray solid masses with whorled appearance on the cut section and a firm to rubbery consistency [11]. Histopathologically, they show low levels of mitotic activity with little to no atypia and necrosis, which are comparable with uterine leiomyoma. Cystic and degenerative changes are more frequent in retroperitoneal than uterine leiomyomas [11]. Thus, based on its morphological aspects, the resected tumor from the patient in this study was categorized as HLM, according to the World Health Organization's classification of the uterine mesenchymal tumor [12].

The treatment of retroperitoneal tumors is challenging, mainly because of their anatomical location, dimensions, and involvement of blood vessels or adjacent organs [13, 14]. Considering the high proportion of malignancies, a well-planned surgery conducted by an experienced oncologist is important. An intraoperative frozen section that provides rapid diagnosis may be helpful in guiding surgical decisions. Surgical excision, via either laparotomy or laparoscopy, is the gold standard treatment for retroperitoneal leiomyoma [15]. Laparoscopic tumor resection was performed on the patient in this study.

This study involving a patient with retroperitoneal HLM was impressive not only because of the rare location of the tumor but also because of its accompanying hydropic changes. Furthermore, the lesion mimicked an ovarian cyst at its initial presentation based on the radiologic features, making it difficult to diagnose accurately until an in-depth histopathological evaluation was performed. By warning of this case, surgeons can recognize the disease entities and provide the necessary treatment.

## Data Availability

The data supporting the findings of this study are available from the corresponding author upon reasonable request.
